# Landscape Scale Influences of Forest Area and Housing Density on House Loss in the 2009 Victorian Bushfires

**DOI:** 10.1371/journal.pone.0073421

**Published:** 2013-08-29

**Authors:** Owen Price, Ross Bradstock

**Affiliations:** Centre for Environmental Risk Management of Bushfires, Institute for Conservation Biology and Environmental Management, University of Wollongong, Wollongong, New South Wales, Australia; NASA Jet Propulsion Laboratory, United States of America

## Abstract

Previous investigations into the factors associated with house loss in wildfires have focused on the house construction and its immediate environment (e.g. gardens). Here, we examine how nearby native forest and other houses can influence house loss. Specifically, we used a sample of 3500 houses affected by the Victorian bushfires of February 7th 2009 to explore how the amount of forest, proportion of forest burned by crown fire and the number of nearby houses affected house loss and how far from the house this influence was exerted. These fires were the most destructive in Australian history and so represent the extreme of fire risk. Using generalized linear modeling we found that the probability of house loss increased with forest extent and the proportion burnt by crown fire and this relationship was strongest for forest measured 1 km from the houses. Houses were more likely to be destroyed if there were other houses within 50 m and if they were on a slope. A model containing these variables predicted house loss with 72% accuracy. Our findings have three important implications: i) management to change the occurrence of crown fire will be effective in reducing house loss; ii) this management may be required up to 1 km away from houses in some situations (a much larger zone than is currently used); iii) high density of houses may increase risk of loss. Given the potentially large width of this management zone and the hazard from nearby houses, it may be more sensible to concentrate on modification of buildings to reduce their vulnerability.

## Introduction

Wildfires pose major risks to people and property (i.e. buildings and other human infrastructure) in many temperate regions of the world [1]. Mitigation of risk is complex and challenging because of the multi-faceted nature of the problem [2,3]. Fires ignite and spread through vegetated landscapes, then reach and encroach into urban environments. Then, factors such as property design and garden configurations further determine the spread and destructive potential of fire. Thus there are multiple possibilities for managed intervention to alter the sequence of processes that determine the probability of losses from fires. These range from actions to reduce ignitions, spread rate and the intensity of fires in landscapes, through to the planning and management of urban development adjacent to the wildland/urban interface (WUI)

Considerable research has been devoted to understanding how attributes of buildings and their immediate surrounds influence the probability of their loss from fires. For example, the design and material composition of the house, the presence of people defending the house, the nature of the garden and the distance from native forest have been found to strongly influence the probability of destruction [4,5,6,7,8,9,10].

While such research addresses the likelihood that a house will burn, given that the fire has arrived at the WUI it does not deal with the way that the condition of the surrounding landscape affects house loss through inherent influences on the initiation and propagation of the fire. Syphard et al [1] found that the dominant fuel type within 1 km of houses has an influence on risk of loss in southern California, but there is little empirical evidence of how the spatial arrangement of vegetation or the intensity of the fire within it actually influences the risk of house loss. It is important to understand these effects because management actions that influence vegetation composition and structure and resultant fire intensity can be taken to reduce the risk. For example use of fuel treatments (e.g. prescribed burning) or the enforcement of appropriate setbacks of buildings from flammable vegetation may ameliorate risk through alteration of fire behavior in the vicinity of houses.

We address this problem by examining patterns of house destruction resulting from fires in *Eucalyptus* dominated forests of Victoria, Australia in February 2009 in terms of potential landscape fuels. These major fires destroyed > 2000 buildings and caused 173 human deaths [11],, and represent the extreme of potential fire intensity. We identified areas of crown fire from a remotely sensed map of fire severity and used measures of crown fire extent around houses to investigate their influence on house loss. Fires that consume the forest canopy burn at high intensity (>10,000 kw m^-1^) and are non-suppressible [12], and are therefore likely to pose a high risk to property. Fire behavior may also be influenced by the proximity of other houses [5,9], which may add fuel to the fire. Therefore, we also measured house density around each house. We used these data to address the questions: i) Is house loss influenced by the proportion of the adjacent forest that experiences a crown fire and/or the density of nearby houses? ii) Over what distance from a house is this influence exerted? iii) What are the relative contributions of vegetation extent, fire severity and house density to house loss? iv) How could the impact be reduced?

## Methods

### Study area

**Figure 1:**
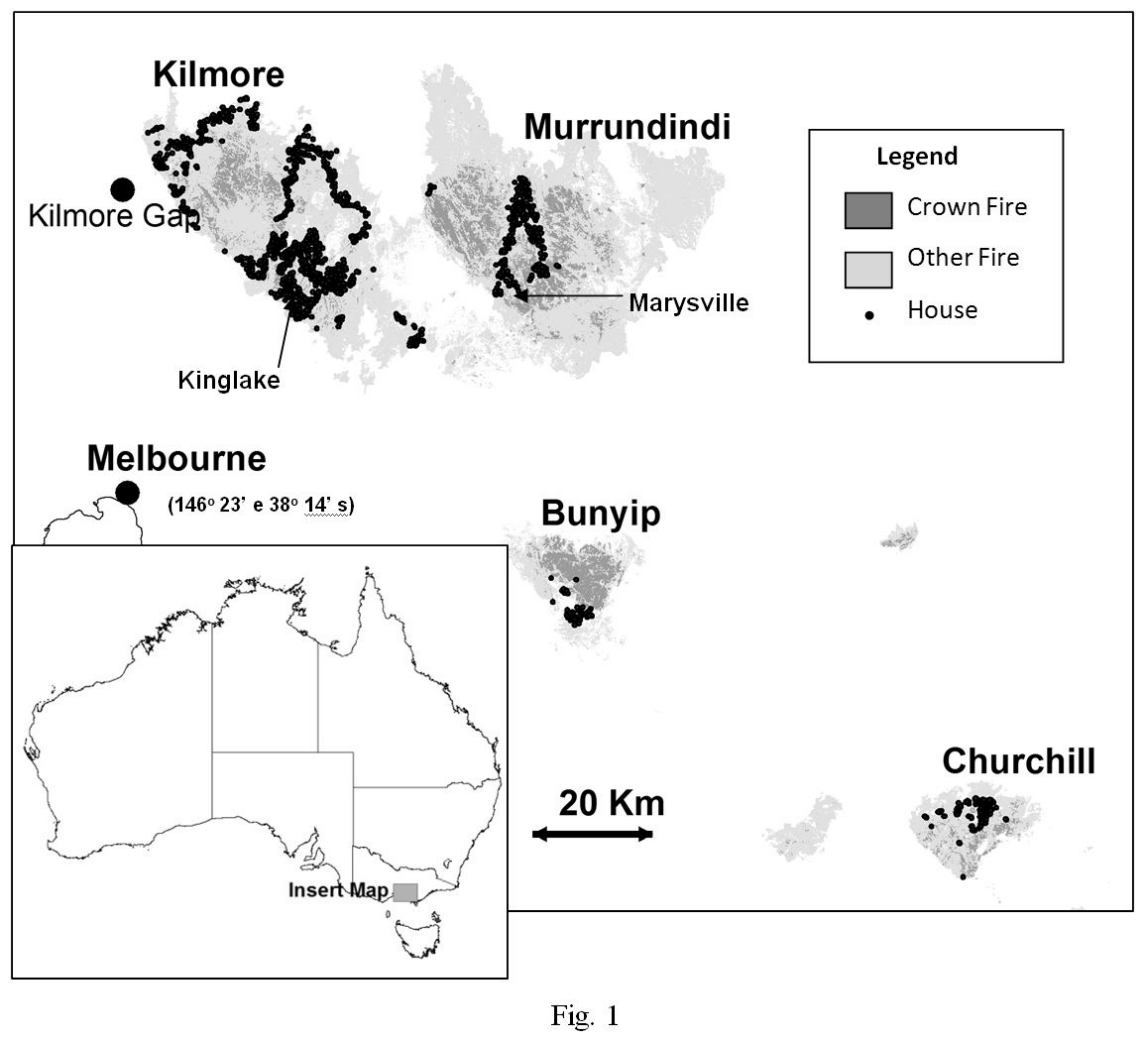
Study area showing areas burnt under crown fire and the locations of houses. The towns of Marysville and Kinglake are also indicated.

Four fires (Kilmore, Murrundindi, Bunyip and Churchill) ignited on February 7^th^ 2009 in southern Victoria were examined (Figure 1). These fires occurred in a matrix of various forest types and land uses, including cleared, agricultural land and *Pinus* plantations. The fires spread rapidly and burned intensely under fire-weather conditions equivalent to the most severe in recorded history [11]; (e.g. 46^o^C, 9% relative humidity, 90 kmh^-1^ wind gusts in many places). At about 17.30, a cold front brought a change from north-easterly to south-westerly winds, an increase in humidity and reduction in temperature across the fire ground. The fires then expanded rapidly from their by-now very long northern flanks, though at a lower intensity than before the southerly change. By about midnight, the conditions had eased, but some fires continued to burn for many more days. The four fires examined here accounted for the great majority of the total area burnt, loss of life and 95% of the properties destroyed in Victoria in 2009.

### Data

The damage to all of the 3518 houses located within the area affected by the four fires was surveyed by the Busfhires Cooperative Research Centre (unpublished data, Fig. **1**). Housing density was estimated as the number of other houses within circles of increasing radius around each house (50 m, 100 m, 200 m, 500 m, 1000 m, 2000 m, and 5000 m). We also estimated the proportion of forest in the landscape surrounding each house using circles of increasing radius (as for house density). The area of forest was defined using 1:100,000 Ecological Vegetation Classes (mapped in 2005, Victorian Department of Sustainability and Environment (DSE), unpublished data), by combining all forest classes. In this region, this definition excludes only modified land (urban or agricultural developments).

Fire severity was mapped by DSE (unpublished data) from SPOT 4 and 5 satellite imagery (10 m resolution). The method applied used a pre- and post-fire difference value in the ratio of near infra-red and short-wave infra-red bands and subsequent classification to match field measurements of severity, following DSE’s published standard operating procedure [13]. We estimated the proportion of the forest that experienced crown fire (severity class 1) in increasing circles corresponding to those used for the forest area and house density calculations.

To control for effects of topography (i.e. prevent them from masking other effects), we incorporated measures of elevation, slope aspect (in four classes) and topographic position for each house into the analysis. These were derived from a 25 m resolution Digital Elevation Model (DSE, unpublished data), the latter by calculating the highest and lowest point within 500 m of every house and then expressing the elevation of the house by its percentage within this range (i.e. local ridge tops are 100 and valley bottoms are 0). Values for these variables were calculated for each of the 3158 houses. The houses were divided into two groups by setting aside the neighbors of each house (unless the neighbor was more than 1 km away). This gave a sample of 1942 houses for analysis, with a further sample of 1576 houses being used to test the accuracy of the selected statistical models.

### Analysis

House loss was analyzed as a binary response: 1 and 0 indicating destroyed and not destroyed houses, respectively. Houses with minor damage were classed with the undestroyed houses. Exploratory analysis indicated that the classification of damaged houses did not have much influence on the results. Models of the probability of house loss in response to environmental attributes and housing density were constructed using binomial regression, a form of generalized linear modeling [14]. Preferred models were selected using Akaike’s Information Criterion (AIC) and a pseudo-r^2^ [15]. Predictor variables selected in the preferred model were tested to ensure that they were not cross-correlated.

For each of the radii (50 m to 10 km), models were fitted to house loss with the area of forest and proportion of crown fire within the forest as the predictors, to identify the distance at which these factors excerpted the strongest influence. The two variables were considered together because their product is a measure of the total fire energy in the area adjacent to houses [16]. The analysis used binomial regression [14]. A similar analysis was conducted using the density of houses as a predictor (i.e. models at each radius).

Then a preferred model was developed that combined forest area, proportion of crown fire, house density and topography as predictors. For this analysis, all combinations of predictors and two way interactions were tested, with the best model and supported alternatives being identified using AIC [17]. The accuracy of the preferred model was estimated by comparing predicted versus actual house loss using the data that was set aside from the analysis and the goodness of fit was measured using a pseudo-r^2^ measure based on log-likelihood [15]. To account for spatial autocorrelation in the data, the analysis was repeated with a spatially lagged response variable [18]: the distance weighted mean destruction of neighboring houses. Also, to reduce possible spatial bias, the analysis was repeated excluding houses from the two largest and most densely packed towns (Marysville and Kinglake, 41% of the total houses). We also repeated the modeling using an upwind wedge of 90^o^ around each house instead of a full circle for each predictor but these were worse predictors of house loss.

The spatial analyses were conducted in ARCMAP 10 Geographic Information System and statistical analyses were performed using R statistical software [19].

## Results

From the total sample of 3518 houses, 1689 were destroyed (48%), and a further 493 suffered minor damage (14%). Forests were the dominant landscape context for these houses: all except one were within 1 km of forest. Crown fires affected only 13% of the forest within 1 km of the houses, the remainder being less severe fire.

**Figure 2:**
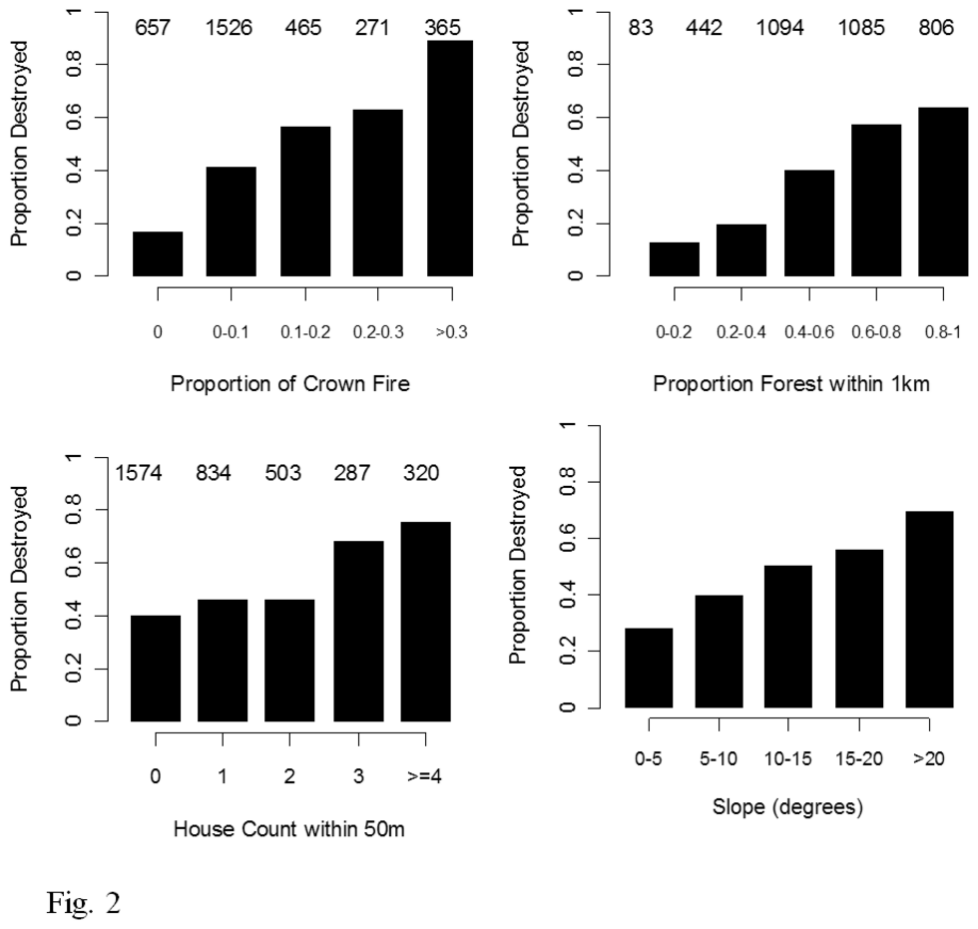
The proportion of houses destroyed for classes of four predictor variables. For each plot, the number of houses in each class is displayed at the top.

The proportion of houses lost was positively related to both the area of adjacent forest and the proportion of crown fire within the adjacent forest (illustrated at 1 km radius in Figure 2). House loss also increased as a positive function of house density and slope (Figure 2).

The goodness of fit of relationships between house loss, forest area and proportion of crown fire peaked at a radius of 1 km (pseudo-r^2^ = 0.21, Table 1). This relationship explained almost three times as much variation in house loss than when measured at radii of 200 m or less. The relationship between neighboring house density and house loss was relatively weak (though statistically significant) with a best radius of 50 m (r^2^ = 0.05) (Table 1).

**Table 1:** Results from binomial regression analysis.

**Radius**	**a) Crown fire and forest area**			**b) House density**		
	ΔAIC	r^2^	p-value	ΔAIC	r^2^	p-value
**50 m**	386.09	0.031	< 2e^-16^	0	0.048	< 2e^-16^
**100 m**	281.82	0.081	< 2e^-16^	15.41	0.041	< 2e^-16^
**200 m**	304.52	0.071	< 2e^-16^	31.16	0.033	< 5e^-15^
**500 m**	28.66	0.194	< 2e^-16^	36.88	0.030	< 2e^-14^
**1000 m**	0	0.206	< 2e^-16^	55.60	0.020	< 2e^-10^
**2000 m**	62.07	0.180	< 2e^-16^	88.35	0.004	0.006
**5000 m**	258.83	0.092	< 2e^-16^	95.49	0.000	0.572

The table shows the effects of a) fire severity and adjacent forested area; and b) house density, calculated at different radii around houses, on house losses from the 2009 Victorian fires. The ΔAIC column shows the difference in Akaike’s Information Criterion between each radius and the best radius, the r^2^ column gives the proportion of variation in house loss explained at that radius (pseudo-r^2^) and the p-value shows the level of statistical significance. The best radius is indicated by ΔAIC value of 0 and the largest r^2^.

**Figure 3:**
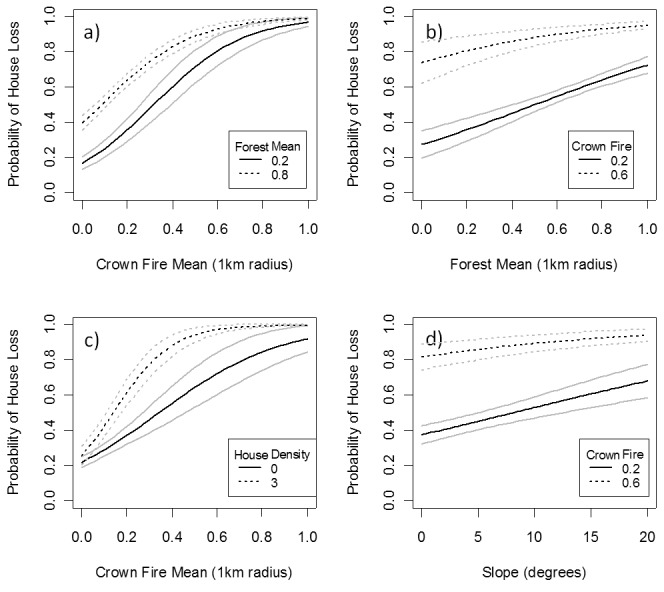
The modeled relationship between likelihood of house loss and predictor variables crown fire, forest area, house density and slope. 95% confidence intervals are shown as grey lines. Predicted effects on house loss of: a) crown fire at quartile levels of forest extent; b) Forest area at quartile levels of crown fire; c) crown fire at two levels of house density; d) Slope at quartile levels of crown fires. For each plot, the unplotted variables are held at their median values.

The preferred combined model for prediction of house loss in relation to crown fire (1000 m radius) contained forest area and slope as main effects and an interaction between house density and proportion of crown fire as significant predictors (Table 2, Figure 3). This model had a pseudo-r^2^ of 0.23 in the training data, and an area under the curve (AUC) of 0.79 and accuracy of 72% for predicting house loss in the test data using a threshold of 0.47 on the model predictions. There were no supported alternative models. None of the selected predictor variables were correlated with each other (maximum correlation = 0.32). House loss was strongly positively related to crown fire and somewhat less so to forest area (a predicted difference in loss probability of 0.7 across the range of crown fire in the data cf 0.4 in forest area). Risk of loss increased with house density (50 m) particularly at intermediate levels of crown fire. These modeled effects were unaffected by spatial autocorrelationor removal of data for the towns of Marysville and Kinglake (Supporting Information S1).

**Table 2:** Model estimates table for the best model of house loss at 1 km radius (n = 1942, pseudo-r^2^ = 0.230).

**Term**	**Estimate**	**Std. Error**	**z value**	**P**
**(Intercept)**	-2.352	0.179	-13.114	0.000
**House density**	0.068	0.051	1.338	0.181
**Crown fire 1k**	3.697	0.547	6.761	0.000
**Forest area 1k**	1.935	0.281	6.895	0.000
**Slope**	0.063	0.013	4.996	0.000
**House densityxCrown fire 1k**	1.317	0.356	3.700	0.000

The estimates column gives the predictive equation l, where probability of house loss = exp(l)/(exp(1+l). The term ‘House density x Crown fire 1k’ is an interaction: the product is used.

## Discussion/Conclusion

The results showed that house loss increased as a positive function of the area of adjacent forest and the proportion of crown fire within these forests and with house density. The effect of house density peaked at a distance of 50 m while that of adjacent forest and crown fire peaked at 1 km. Overall these effects reinforce the notion that separation of property from flammable native forest is required to effectively reduce risk of loss.

The chance of destruction increased with house density at a scale similar to that found by Gibbons et al. [9] (i.e. circa. 50 m). This result implies that risk is posed by houses that are close (i.e. immediate neighbors) but not those that are distant. Neighboring houses and forest therefore act as fuel that can potentially pose a threat to any particular house if ignited, but the distance over which these influences are exerted is different. The 50 m influence of houses is approximately the zone of flame contact or high exposure to radiant heat. For example, 199 densely packed houses were lost in the Grass Valley Fire (2007, Lake Arrowhead, California, USA), due to house-to-house spread [7]. Cohen [20] defined the Home Ignition Zone as the area between 30 and 60 m around the house that directly influences ignition of the house. Our results validate this concept. Syphard et al. [1] found that risk of house in southern California was highest at low and intermediate housing densities, which appears contrary to our positive relationship with housing density. However, by analyzing only houses within the fire perimeter, we excluded high density suburbs with very low fire risk that Syphard et al. [1] included. Had we used their approach and included unaffected nearby cities such as Melbourne we would probably have found the same effect. The highest house density in our study was 9 houses/ha, which is relatively low density. The important conclusion from our study is that nearby houses can be an additional risk factor.

The 1 km influence of forests is well beyond the flame zone and combined with the strong positive effect of crown fire this suggests that the main source of hazard from the forest is embers. Embers or firebrands can on occasion be carried for several kilometers from intense fires [21,22]. Some houses destroyed in the 2009 Victorian fires were more than 380 m from forest blocks [10]. Loss of houses at similar distances (i.e. 300 to 700 m) from the edge of eucalypt forests has also been recorded in other major Australian fires [23,24]. These distances are much larger than those specified as fuel-free or low fuel setbacks by fire management authorities in relevant Australian jurisdictions (e.g. 25 to 500 m). This suggests that current management approaches are inadequate to cope with the conditions experienced in these fires. This does not necessarily imply that such large distances are involved in other fires around Australia or more broadly. These forests have very high fuel loads and the weather was exceptionally bad. Our results probably represent the upper limit of the distance of influence of native vegetation. Further research is required to determine the relevant scales in other situations.

House density and crown fire extent act in synergy. The presence of houses boosts the crown fire effect on house loss and the presence of high levels of crown fire boosts the house density effect on house loss. This suggests that there may be some feedback between them, perhaps increasing the overall energy output of the fire.

While the combined model of house loss gave accurate predictions of house loss (>70% correct), the majority of the variation in house loss remained unexplained. This is probably due to the characteristics of the houses and their gardens. Gibbons et al. [9] analyzed the factors in the immediate vicinity of houses in these fires and found that the amount of vegetation within 40 m of the house had a strong influence on house loss, and it was higher if the vegetation was native rather than planted. Also, the likelihood of destruction, is influenced by factors such as construction material, the presence of people and the actions, and trees overhanging roofs [10]. These factors were not included in our analysis. It should be possible to combine our landscape scale analysis with these fine-scale variables to obtain a more comprehensive understanding of the factors causing house loss.

Overall, the results reinforce the notion that forests act to bring a fire to urban areas whereas houses promote transmission of fire within developments once a fire has arrived [25]. Our findings indicate that the magnitude of separation of houses from flammable forests that is required for their effective protection is potentially very large (e.g. circa. 1 km). Importantly, the area of forest within 1 km had more influence than the area of forest within 100 or 200 m of the house. This distance reflects a balance between the quadratic scaling of area with distance and the attenuation of influence of energy and ember output. The scaling implies that if 25% of the forest within 1 km of a house experiences crown fire, the total energy and ember output is 12 times greater than if 50% of the forest within 200 m experiences crown fire (i.e. energy ~ 0.25 * 1000^2^ = 250,000, c.f. 0.5 * 200^2^ = 20,000). We also analyzed the variables driving the proportion of crown fire and found strong positive effects of weather and forest area within 1 km (Supporting Information S2). This indirect effect of forest area on house loss is presumably also a scaling phenomenon: the larger the area of forest that burns, the more likely that it will burn at high intensity. Countering the scaling effects, radiant heat decreases with distance from the fire source, as reflected in the 40 m effect of vegetation found by Gibbons et al. [9]. The large distance here probably reflects a dominant role of ember production compared to radiant heat. The critical distance at which forest vegetation must be managed to mitigate risks from fire identified in our study is therefore circa. 1 km. This may be achieved either by reducing the amount of forest or reducing the likelihood of crown fire through treatment of key fuel elements such as surface litter [12].

Management must therefore address the area of the forest (i.e. total clearance), the factors promoting crown fire and the placement of houses. These findings pose major challenges. Clearance of forest up to 1 km from houses is unlikely to be acceptable to the wider community because of potential environmental consequences and degradation of aesthetic values, though major new developments could be suitably planned. An increase in fuel treatment, such as prescribed burning may reduce crown fire risk but it has also been shown that fire severity in these fires was not reduced by recent burning (reduced fuel) under very severe weather [26]. In any case very high treatment rates may be needed to significantly reduce risk and this may pose challenges in terms of cost [27] and environmental effects [28]. Increased emphasis on planning new developments with suitable setbacks (to forest and other houses) and construction standards as well as improvement to the condition of the existing stock of houses and their gardens may be desirable. This would have the dual benefit of directly reducing the likelihood of loss and reducing the role of houses as fuel to burn other houses. Also, discussion of possible strategies must acknowledge that these were the most damaging fires in Australian history and hence question whether it is appropriate to apply drastic management actions to address risks that occur so rarely. Considered answers to these questions require full quantification of risk (likelihood versus consequence).

## Supporting Information

Supporting Information S1 Addressing spatial bias and autocorrelation.
(DOCX)

Supporting Information S2 **Analysis of drivers of crown fire proportion.**
(DOCX)
