# Supplementary material for: Landscape Scale Influences of Forest Area and Housing Density on House Loss in the 2009 Victorian Bushfires
Source: PLoS One. 2013 Aug 29;8(8):e73421. doi: 10.1371/journal.pone.0073421 (PMC3756981; doi:10.1371/journal.pone.0073421)
Supplement: Supporting Information S1 — Addressing spatial bias and autocorrelation. (DOCX) [file pone.0073421.s001.docx]

**S1: Addressing spatial bias and autocorrelation**

To account for spatial autocorrelation in the data, a best model of house loss was developed that combined forest area, proportion of crown fire, house density and slope as predictors plus a spatially lagged response variable ([Haining 2003](#_ENREF_1)): the distance weighted mean destruction of neighboring houses. Also, to reduce possible spatial bias, the analysis was repeated excluding houses from the two largest towns (Marysville and Kinglake, 41% of the total houses).

When the distance weighted mean of neighbouring house loss was included in the modelling, the best model was the same as when it was not included, except that the strength of the relationships for House density and crown fire were weaker (lower estimates) (Table S1). When houses from the towns of Marysville and Kinglake were excluded from the modelling, the best model contained the same terms as the model including those towns, and the estimates for the terms were also similar (Table S2).

Haining, R (2003) 'Spatial Data Analysis.' (Cambridge University Press: Cambridge)

Table S1. Preferred model including neighbor loss (n = 1942, deviance =466, pseudo-r2 = 0.290, no supported alternatives).

|  | **Estimate** | **Std. Error** | **z value** | **Pr(>\|z\|)** |
| --- | --- | --- | --- | --- |
| **(Intercept)** | -2.724 | 0.191 | -14.270 | < 2e-16 |
| **House density** | 0.033 | 0.054 | 0.606 | 0.545 |
| **Crown fire 1k** | 2.125 | 0.575 | 3.693 | 0.000 |
| **Forest area 1k** | 1.194 | 0.299 | 4.000 | 0.000 |
| **Slope** | 0.053 | 0.013 | 4.025 | 0.000 |
| **Neighbor loss** | 2.439 | 0.206 | 11.824 | < 2e-16 |
| **House density:Crown fire 1k** | 0.946 | 0.342 | 2.762 | 0.006 |

Table S2. Preferred model excluding Marysville and Kinglake (n = 1206, deviance =184.0, pseudo-r^2^ = 0.142, no supported alternatives)

|  | **Estimate** | **Std. Error** | **z value** | **Pr(>\|z\|** |
| --- | --- | --- | --- | --- |
| **(Intercept)** | -2.159 | 0.204 | -10.609 | < 2e-16 |
| **House density** | -0.010 | 0.060 | -0.163 | 0.871 |
| **Crown fire 1k** | 2.466 | 0.719 | 3.431 | 0.001 |
| **Forest area 1k** | 2.129 | 0.314 | 6.782 | 0.000 |
| **Slope** | 0.048 | 0.014 | 3.349 | 0.001 |
| **House density:Crown fire 1k** | 1.610 | 0.604 | 2.667 | 0.008 |
