# Supplementary material for: Landscape Scale Influences of Forest Area and Housing Density on House Loss in the 2009 Victorian Bushfires
Source: PLoS One. 2013 Aug 29;8(8):e73421. doi: 10.1371/journal.pone.0073421 (PMC3756981; doi:10.1371/journal.pone.0073421)
Supplement: Supporting Information S2 — Analysis of drivers of crown fire proportion. (DOCX) [file pone.0073421.s002.docx]

**S2: Analysis of drivers of crown fire proportion**

Given that the degree to which high intensity crown fires may influence house loss, it is important to understand the determinants of these fires. Key influences include weather, topography, fuel load and the vertical arrangement of fuel. Prescribed burning is a common fuel treatment used to alter the amount and structure of fuels and therefore reduce the risk of asset loss and aid the suppression of fires ([Fernandes and Botelho 2003](#_ENREF_2)). We therefore also analyzed the relative influence of fuel age and the other factors on the propensity for crown fires. Specifically we predicted that crown fire propensity was negatively related to the extent of recent burning.

Digital fire history mapping supplied by DSE (unpublished data) was used to derive a measure of fuel age (time since last fire) in the vicinity of the houses just before February 2009. We used the proportion of the landscape burnt in the last five years (termed ‘recent fire’) as the key indicator, because fuel ages within this range are known to have the most substantial effect on fire intensity in eucalypt forests ([Price and Bradstock 2012](#_ENREF_5)).

Within the four fires, areas burnt under four contrasting classes of fire weather were delineated, using fire progression maps (DSE unpublished data), cross-referenced against half-hourly weather records from Bureau of Meteorology (BOM) weather stations ([Price and Bradstock 2012](#_ENREF_5)). They were classified according to the revised Australian Fire Danger Rating (AFDR) ([Anon 2009](#_ENREF_1)) class as measured from the weather station at Kilmore Gap (Fig. S1). The revised AFDR system defines classes according to increasing levels of the Forest Fire Danger Index ([Noble *et al.* 1980](#_ENREF_4)). The periods were: 1) Catastrophic (the period prior the southerly change on February 7^th^, mean FFDI = 103); 2) Very High (the period during which the southerly change occurred so the actual weather experienced was not certain, mean FFDI = 36); 3) Moderate (the period after the southerly change, but before midnight on February 7^th^, mean FFDI = 9), and; 4) Low (days subsequent to February 7^th^, mean FFDI = 3). The use of broad weather classes compared to actual weather at each house was necessary because the precise weather at the time of fire encroachment and destruction was unknown and difficult to accurately estimate.


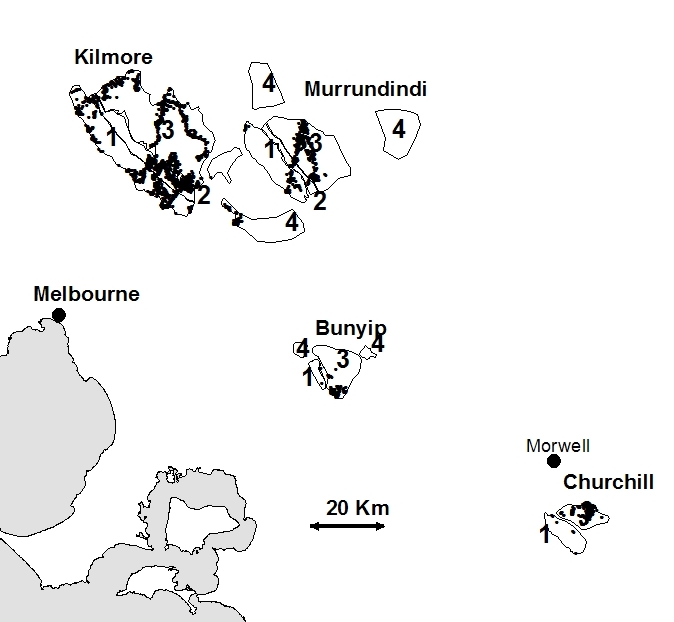

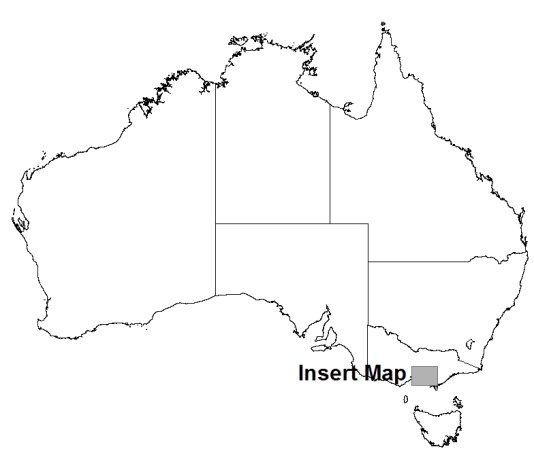


**(146^o^ 23’ e 38^o^ 14’ n)**

**(146^o^ 23’ e 38^o^ 14’ n)**

**Kinglake**

**Marysville**

Figure S1: Study area showing the fires with weather periods and the locations of houses (black dots). The towns of Marysville and Kinglake are also indicated

These analyses were conducted using the 1000 m radius of influence of forest identified by the house loss analyses (i.e. all of the variables were calculated at this radius). These models used a pseudo-binomial response and logit link function because the dependent variable was a proportion.

The best model to predict the proportion of crown fire within 1000 m of houses contained weather class, wind aspect, and a two way interaction between forest area and recent burning (Table S3, Figure S2). The model had a pseudo-r^2^ of 0.34. Forest area had a strong positive effect on crown fire. Weather also had a strong effect such that under low weather conditions, crown fire was very low irrespective of the other variables (e.g. even with high forest area). Recent fire had a positive effect on crown fire, and this was more pronounced when the forest extent was low. The wind-aspect effect was mild and positive, so the probability of crown fire was higher in the lee side of hills than the windward side. This is the same effect as reported by Price and Bradstock ([2012](#_ENREF_5)) for the occurrence of crown fire in the broader landscape in these fires, and may be due to wind vortices on lee slopes that increase fire intensity ([Sharples *et al.* 2010](#_ENREF_6)).

House loss also increased with recent burning at 1000 m radii (Figure S1). Ultimately, there was not enough previous fire in the area to make firm conclusions about its affect on reducing risk (i.e. only 8% of houses had at least 20% of forest recently burnt within 1000 m). However, it is clear that recent burning did not provide a high level of protection from destruction. It is also possible that prescribed burning is focused on areas of known fire risk in this region, which could cause an apparent positive correlation between past fire and subsequent fire. Evidence from previous analysis of severity suggests that previous burning does reduce severity, but not during extremes of weather (either severe or above or low) ([Price and Bradstock 2012](#_ENREF_5)). Also, Gibbons et al. ([2012](#_ENREF_3))found for a subset of the houses examined here, that they were less likely to be destroyed if they were close to areas upwind that were burnt within five years. However that relationship was weak (explaining about 3% of variation in house loss).

Anon (2009) 'Fire Danger Rating.' (Rural Fire Service NSW: Sydney)

Fernandes, PM, Botelho, HS (2003) A review of prescribed burning effectiveness in fire hazard reduction. *International Journal of Wildland Fire* **12**, 117-128.

Gibbons, P, van Bommel, L, Gill, AM, Cary, GJ, Driscoll, DA, Bradstock, RA, Knight, E, Moritz, MA, Stephens, SL, Lindenmayer, DB (2012) Land Management Practices Associated with House Loss in Wildfires. *PLOS one* **7**, e29212.

Noble, IR, Bary, GAV, Gill, AM (1980) McArthur's fire-danger meters expressed as equations. *Australian Journal of Ecology* **5**, 201-203.

Price, OF, Bradstock, R (2012) The efficacy of fuel treatment in mitigating property loss during wildfires: insights from analysis of the severity of the catastrophic fires in 2009 in Victoria, Australia. *Journal of Environmental Management* **113**, 146-157.

Sharples, JJ, McRae, RHD, Weber, RO (2010) Wind characteristics over complex terrain with implications for bushfire risk management. *Environmental Modelling & Software* **25**, 1099-1120.

Table S3: Model estimates table for the best model of predictors of crown fire proportion.

|  | **Estimate** | **Std. Error** | **t value** | **Pr(>\|t\|)** |
| --- | --- | --- | --- | --- |
| **(Intercept)** | -3.964 | 0.164 | -24.199 | 0.000 |
| **Forest area1k** | 2.700 | 0.187 | 14.482 | 0.000 |
| **Weather Mod** | -0.536 | 0.084 | -6.358 | 0.000 |
| **Low** | -4.713 | 1.563 | -3.016 | 0.003 |
| **V. High** | -0.005 | 0.094 | -0.052 | 0.958 |
| **Recent fire 1k** | 13.960 | 1.586 | 8.803 | 0.000 |
| **Wind aspect** | 0.0034 | 0.001 | 5.915 | 0.000 |
| **Forest area 1k:Recent fire 1k** | -13.460 | 2.430 | -5.538 | 0.000 |

Figure S2: The modelled relationship between the proportion of crown fire (at 1 km radius) and predictor variables forest area, weather, recent fire, and wind aspect. 95% confidence intervals are shown as grey lines. Predicted effects on crown fire: a) Forest area for different weather classes; b) Recent burning for quartile levels of forest extent; c) Wind aspect for quartile levels of forest extent. For each plot, the unplotted variables are held at their median values.

a)

b)

c)
